# Effects of Fan Noise on Growth Performance, Blood Parameters, Feeding Behavior, and Slaughter Performance of Geese Aged 21–70 Days

**DOI:** 10.3390/ani16071039

**Published:** 2026-03-28

**Authors:** Qun Xie, Xiaofeng Huang, Zuolan Liu, Ying Chen, Yue He, Xinyu Chang, Qiang Cheng, Guangliang Gao, Yi Luo, Haiwei Wang, Qigui Wang, Jiajia Xue, Chao Wang

**Affiliations:** Poultry Science Institute, Chongqing Academy of Animal Sciences, Rongchang, Chongqing 402460, China; xiequn20221208@163.com (Q.X.); hxf2007@yeah.net (X.H.); liuzuolan9229@163.com (Z.L.); chenying.cq@163.com (Y.C.); heyueh@163.com (Y.H.); changxinyu1216@163.com (X.C.); chengqiangcool@163.com (Q.C.); gaogl@cqaa.cn (G.G.); homleestar@163.com (Y.L.); wanghw@cqaa.cn (H.W.); wangqigui@hotmail.com (Q.W.)

**Keywords:** fan noise, geese, growth performance, stress, antioxidant status, feeding behavior

## Abstract

We conducted this experiment with the aim of investigating the effects of different noise levels from ventilation fans on the growth and slaughter performance, meat quality, blood parameters, and feeding behavior of geese from 21 to 70 days of age. A total of 108 male geese (21-day-old) were randomly assigned to one of three conditions: a control group (no additional fan noise), low-noise treatment (65–75 dB), and high-noise treatment (85–95 dB). Each treatment included six replicates, with six geese per replicate. The results showed that neither ventilation fan noise level significantly affected growth performance, feeding behavior, slaughter performance, or major meat quality traits (*p* > 0.05). Compared with the control group, noise exposure significantly reduced circulating adrenocorticotropic hormone and corticosterone concentrations (*p* < 0.05), and the low-noise group exhibited significantly reduced cortisol concentrations (*p* < 0.05), while the high-noise group had increased cortisol concentrations. Under noise exposure conditions, no statistically significant effects were observed on superoxide dismutase, total antioxidant capacity, malondialdehyde concentration, catalase, and glutathione peroxidase activities compared with the control group (*p* > 0.05). Overall, prolonged noise stimulation (65–75 dB and 85–95 dB) alleviated stress responses in commercial geese aged 21–70 days, without negatively affecting their growth performance, slaughter performance, meat quality, or feeding behavior.

## Simple Summary

Currently, ventilation fans are the core equipment used for environmental control in large-scale farms, offering numerous advantages, such as improving the poultry house environment and reducing mortality rates. However, ventilation fans generate significant noise during operation, and geese are naturally vigilant and highly susceptible to stress caused by sound disturbances. Hence, this study was conducted to investigate the effects of prolonged, steady fan noise in growing geese. The results found that long-term exposure to fan noise can alleviate the stress responses in geese and has no negative impact on the growth performance, slaughter performance, major meat quality traits, or feeding behavior of geese aged 21 to 70 days.

## 1. Introduction

To improve on-farm conditions, modern livestock production is rapidly shifting toward greater mechanization and digitalization. In poultry operations, deploying ventilation infrastructure and automated feeding systems has improved environmental control while reducing labor intensity [1,2,3,4]. However, with the continuous advancement of the livestock production intellectualization level, the issue of noise pollution within farms has become increasingly prominent.

Existing research has produced extensive findings on the impacts of noise on poultry, with some studies on the one hand finding that noise stimulation can have adverse effects on poultry. For example, 65–75 dB noise stimulation could cause a continuous inflammatory response in laying hens, which reduces the immunity and antioxidant capacity of the body, leading to a decline in the laying hen welfare level [5]. Subway noise stimulation inhibited the development of ovarian follicles and suppressed the formation of secondary follicles in 100-day-old hens [6]. Acute noise exposure can induce splenic damage in chickens via oxidative stress, inflammation, and apoptosis by modulating the Keap-1/Nrf2 and NF-κB pathways [7]. Additionally, high-intensity noise has been shown to induce stress in poultry, because a specific noise stimulus (90 dB versus 65 dB) causes stress and fear in laying hens [8]. A brief 10 min noise episode can significantly elevate serum corticosterone concentrations in chickens [9].

On the other hand, the effects of noise are not exclusively negative. Some studies have reported that noise stimulation exerts certain positive effects on poultry; for example, prolonged exposure to livestock farm environmental sounds at different intensities (65–75 dB and 85–95 dB) promoted the development of the intestinal barrier and the colonization of beneficial microbiota in pullets [10]. Prolonged exposure to music or noise at varying decibel levels effectively mitigated the negative impact of transportation stress on pullets, and sound stimuli with high decibel levels (ranging from 85 to 95 dB) demonstrated greater potential in alleviating transport-induced stress [11]. Moreover, it was found that loud noise at 90 dB in commercial hatcheries could be beneficial for incubation, resulting in earlier hatching, higher hatchability, and better chick quality [12].

Existing research on the effects of noise on poultry has largely focused on chickens, while studies on its impact on geese are very limited and this subject is largely unexplored. Currently, the shift in the commercial geese farming model from traditional free-range to large-scale farms has increased reliance on ventilation, which is essential for regulating the indoor environment, especially during the hot, humid summers in southern China. Ventilation fans have numerous advantages, such as improving the goose house environment and reducing mortality rates; however, they generate significant and persistent noise during operation. During normal ventilation fan operation, we measured the noise intensity inside the goose house (in the absence of geese) and found that noise levels in cages near the ventilation fans exceeded 80 dB, consistent with noise levels reported in some chicken farms. For example, Oh, T.K. et al. [13] discovered maximum and average noise intensities of 90 dB and 69 dB, respectively, in laying hen farms with mechanical ventilation. Liu Keling [14] measured noise intensity in a cage-reared hen house equipped with a ventilation system and found that it reached 82.3 dB at a distance of 1 m from the ventilation system. This indicates that geese of large-scale farms are routinely exposed to moderate-to-high-intensity noise throughout the rearing period. Geese possess a well-developed sense of hearing, which makes them particularly responsive to acoustic changes in their surroundings, and they are naturally vigilant and highly susceptible to stress caused by sound disturbances; however, the effects of prolonged ventilation fan noise on geese remain largely unexplored. Therefore, referring to the existing research on the effects of noise on chickens, in this study, we focused on commercial male geese aged 21 to 70 days to investigate the effects of prolonged, steady ventilation fan noise on their growth performance, blood parameters, feeding behavior, and slaughter performance. The objective was to determine whether ventilation fan noise induced adverse effects in geese and to provide references for poultry farming practices.

## 2. Materials and Methods

### 2.1. Experimental Management and Procedures

All animal care and experimental procedures in this study were performed in accordance with the Regulations for the Administration of Affairs Concerning Experimental Animals of the People’s Republic of China and approved by the Animal Care and Welfare Committee of the Chongqing Academy of Animal Science (CAAS), China (approval number XKY-202211B01). All geese used in this study were obtained from the CAAS goose-breeding center.

A total of 108 commercial male Sichuan White geese (21 days old) with similar initial weight were studied. Geese were randomly assigned into three treatments: a control group (no fan noise; loudness 0 dB), a low-noise group (fan noise; loudness 65–75 dB), and a high-noise group (fan noise; loudness 85–95 dB). Each treatment consisted of six replicates with six geese per replicate. After group assignment, geese were housed separately in three identical goose houses within the same farm under otherwise identical rearing conditions. Geese were kept in large cages and received the same diet in all treatments; pelleted feed and water were provided ad libitum. Stocking density was 0.3 m^2^ per goose. The experiment lasted 49 days.

The noise was recorded using the SoundTestV1.0 noise monitoring system, part of the TS-MNS sound acquisition system (Beijing Huaqingsi Chuangsheng Technology Co., Ltd., Beijing, China). The system includes PC-based software, a professional acquisition card, a power amplifier, a measurement microphone, and a calibrator. A record-and-playback method was employed: the ventilation fan noise was first recorded and then played back through an audio system, with the playback intensity adjusted as needed. During recording, the sensor (TS-MNP21; Beijing Huaqingsi Chuangsheng Technology Co., Ltd., Beijing, China) was placed 1 m from the ventilation fan and oriented toward the center of the ventilation fan shaft. A 5 min audio segment was recorded, saved to a computer via monitoring software, and transferred to a USB drive for playback through an audio system. In the noise exposure treatments, a USB-connected loudspeaker was positioned 1 m above the rearing pens. A handheld sound level meter (Delixi; Delixi Electric Co., Ltd, Yueqing, China) was used to measure the noise intensity inside the goose house. Five measurement points were randomly selected in each noise treatment group’s pen. The sound level meter was positioned at the height of the geese’s heads to measure noise intensity. Each point was measured three times, with readings taken at 1 min intervals, and the average value was calculated. During the measurement, the volume of the played noise audio was adjusted to ensure that the average noise intensity at each measurement point fell within the range of 65–75 dB for the low-noise group and 85–95 dB for the high-noise group. The recorded noise track was then played on a continuous loop each day from 10:00 to 18:00 throughout the experimental period.

### 2.2. Sample Collection and Measurement of Outcomes

#### 2.2.1. Growth Performance

Initial body weight (IBW) was recorded at baseline (21 days of age) for each replicate of each treatment group. At the end of the trial (70 days of age), geese were reweighed, and residual feed was collected and weighed. Throughout the experimental period, the number of deaths per replicate and the amount of feed added were recorded. The final body weight (FBW) was measured after a 12 h fasting period (with water available) using an electronic scale (YH-T1, Yingheng, Huizhou, China; capacity: 70 kg, accuracy: 1 g). Based on these data and considering the body weight gain of the dead geese, the average daily gain (ADG), average daily feed intake (ADFI), feed-to-gain ratio (F/G), total feed intake (TFI), and mortality were calculated.

#### 2.2.2. Slaughter Performance

At the end of the experiment (70 days of age), one goose per replicate with body weight close to the replicate mean was selected to record live weight. After exsanguination and defeathering, Carcass weight and semi-eviscerated and fully eviscerated weights were measured. The heart, liver, abdominal fat (including fat surrounding the gizzard), spleen, and testes were collected and weighed individually. Breast and leg muscles were then dissected and weighed. Dressing percentage, breast and leg muscle yields, skin-plus-subcutaneous-fat yield, and abdominal fat yield were computed following Terminology and Measurement Methods for Poultry Production Performance (NY/T 823-2020 [15]).

#### 2.2.3. Meat Quality

Samples of the right breast muscles were collected at 45 min post-slaughter and stored at 4 °C for color and pH analysis. Three locations per sample were evaluated using an automatic colorimeter (DP-400; Konica Minolta, Tokyo, Japan). The average of the three readings was used as the color value for each sample. Color was summarized using the CIE Lab* system: lightness (L*), redness (a*), and yellowness (b*).

The pH of breast muscles was measured using a portable pH meter (TESTO-205; Testo SE & Co. KGaA, Titisee-Neustadt, Germany).

#### 2.2.4. Blood Parameters

At 70 days of age, blood samples were collected from 6 geese with a weight close to the average weight of the treatment (each treatment was represented by 6 replicates, with one goose per replicate) after a 12 h fasting period. Approximately 5 mL of blood was drawn from the jugular vein using anticoagulant vacuum tubes. Plasma was separated by means of centrifugation (3500 rpm, 15 min, 4 °C; 5424R, Eppendorf, Shanghai, China) and stored at −80 °C for antioxidant and hormone analysis. The total antioxidant capacity (T-AOC), content of malondialdehyde (MDA), and activity of glutathione peroxidase (GSH-Px), superoxide dismutase (SOD), and catalase (CAT), and levels of adrenocorticotropic hormone (ACTH), corticosterone (CORT), and cortisol (COR) in the plasma were detected using commercial analytical ELISA kits according to the manufacturer’s recommendations (T-AOC, A015-1-1; MDA, A003-1-1; GSH-Px, A005-1-1; SOD, A001-1-1; CAT, A007-1-1; ACTH, H097-1-1; CORT, H205-1-1; COR, H094-1-1; Nanjing Jian cheng Bioengineering Institute, Nanjing, China).

#### 2.2.5. Feeding Behavior

Feeding behavior was analyzed using video recordings obtained from high-definition cameras installed on the ceiling of each treatment house, ensuring complete visual coverage of all replicates. During the trial, video footage from three randomly selected days (excluding days with extreme weather or management interventions) was used for behavioral quantification. Observations were conducted during two daily windows (11:00–13:00 and 15:00–17:00) to capture peak feeding activity. For each observation window, feeding behavior was scored using instantaneous scan sampling at 5 s intervals. Specifically, at each 5 s interval, the number of geese engaged in feeding (defined as the head being inside the feeder or pecking at feed) was recorded. The following parameters were then calculated for each replicate: total occurrences of feeding behavior (the sum of geese observed feeding across all scan samples) and total duration of feeding behavior (estimated by multiplying the number of scans in which feeding was observed by the 5 s interval, assuming feeding behavior persisted throughout the interval). Finally, per-goose averages for feeding frequency (occurrences per goose) and feeding duration (seconds per goose) during the daily observation period were computed by dividing the total occurrences or duration by the number of geese in each replicate. All behavioral recordings were performed by a single trained observer to maintain consistency.

### 2.3. Statistical Analysis

All data were entered and summarized in Microsoft Excel 2016. Statistical analyses were conducted in SPSS 31.0 using one-way analysis of variance (ANOVA). Homogeneity of variances was assessed using Bartlett’s test, and mean differences were evaluated using Tukey’s least significant difference (HSD) procedure. Results are reported as means, and statistical significance is defined as *p* < 0.05.

## 3. Results

### 3.1. Growth Performance

The effects of the ventilation fan noise level on the growth performance of the geese are presented in Table 1. A total of 108 male geese born in the same batch were randomly assigned into three treatments: a high-noise group, low-noise group, and control group. Each treatment included six replicates of six geese. At 21 days of age, no significant differences were observed in the initial body weight of geese among all treatment groups (*p* > 0.05). During the experimental period, stable ventilation fan noises at different loudness levels were applied. At 70 days of age, no significant differences were found among all treatment groups in terms of the final body weight (FBW), average daily gain (ADG), average daily feed intake (ADFI), feed-to-weight ratio (F/G), total feed intake (TFI), or mortality (*p* > 0.05).

### 3.2. Slaughter Performance

Table 2 reports slaughter performance outcomes at 70 days of age. Noise exposure at different loudness levels did not significantly affect dressing percentage, fully eviscerated yield, semi-eviscerated yield, breast and leg muscle yields, skin-plus-subcutaneous-fat yield, abdominal fat yield, or lean meat yield (*p* > 0.05). This indicated that prolonged exposure to ventilation fan noise stimulation did not negatively affect the slaughter performance or final meat production capacity of geese.

### 3.3. Meat Quality

Table 3 summarizes the effects of ventilation fan noise exposure on physical indicators of meat quality at 70 days of age. No statistically significant treatment differences in pH or color traits (L*, a*, and b*) were found for breast muscle (*p* > 0.05). This indicated that prolonged exposure to ventilation fan noise stimulation did not negatively affect the meat quality of geese.

### 3.4. Blood Stress Biomarkers

As reported in Table 4, the geese that were exposed to prolonged ventilation fan noise showed lower plasma ACTH and CORT levels compared with the control group (*p* < 0.05). Among the noise-treated groups, there was no significant difference in ACTH levels, while the concentrations of CORT and COR in the low-noise group were significantly lower than those in the high-noise group (*p* < 0.05). The geese experiencing prolonged exposure to low fan noise showed lower plasma COR levels compared with the control group (*p* < 0.05). By contrast, COR did not differ significantly between the high-noise group and the control group, but COR levels were higher in the high-noise group than in the control group. This result indicates that a prolonged high fan noise environment effectively reduces the physiological stress levels of geese.

### 3.5. Blood Antioxidant Indices

As shown in Table 5, in terms of blood antioxidant indices of the geese, although the SOD activity in the noise-treated groups was higher than that in the control group, the difference was not statistically significant, and SOD activity did not differ significantly between the two noise treatment groups (*p* > 0.05). No statistically significant differences in T-AOC, CAT, GSH-Px activities, or MDA concentration were found among all treatment groups (*p* > 0.05).

### 3.6. Feeding Behavior

As shown in Table 6, ventilation fan noise exposure had no statistically significant effects on feeding behavior in geese aged 21–70 days. In particular, mean daily feeding frequency and feeding duration do not differ across treatment groups (*p* > 0.05). This suggested that prolonged exposure to ventilation fan noise did not alter the basic feeding behavior of geese, nor did it induce significant anorexia or agitation.

## 4. Discussion

### 4.1. Growth Performance

In the present study, we found no significant differences among all treatment groups in terms of growth performance. This seems to not be entirely consistent with previous studies on livestock and poultry, wherein lambs exposed to 75 dB of prolonged noise showed increased average daily weight gain and improved feed efficiency compared to the control and 100 dB groups [16]. Noise as high as 80 dB increased the feed intake of dairy cows, while high-intensity noise (105 dB) decreased their feed consumption and milk yield [17]. Broiler chickens at 1d were exposed to 110 dB intermittent aircraft noise for nine consecutive weeks, with exposure occurring for 5 min every 20 min daily, and showed no significant difference in growth compared to the control group [13]. The exposure of 1-day-old broilers to fan noise (70 or 80 dB) for 7 days significantly decreased live body weight compared to the control group [18], while playing broilers classical music (65–75 dB) increased their ADG and decreased their F/G [19]. However, the findings of these studies indicate that animals have a certain adaptability to sounds, and so we need to find the appropriate sound parameters that different animals can adapt to or produce positive effects in the presence of. Of course, these parameters include not only noise intensity (dB), frequency (Hz), and duration and pattern, but also the hearing ability of the animal species and breeds, the age, the physiological state of the animal at the time of exposure, and the noise exposure history of the animal [20].

Researchers observed that birds showed the greatest increase in auditory threshold in higher-frequency ranges after exposure to 95–100 dB noise [21]. The geese in this experiment seemed to have adapted to the noise environment, as no significant negative impact on growth performance was observed. This may be linked to a reduction in other environmental stressors, such as human entry into the goose house. The decrease in stress indicator levels observed in this trial may also support this perspective.

### 4.2. Slaughter Performance and Meat Quality

There has been little examination of the effect of exposure to ventilation fan noise on goose health and meat quality. Mikołajczak et al. [22] found that wind turbines generate audible noise and infrasound, which may affect the level of stress in animals and consequently their meat quality. A previous study showed the relationship between cortisol levels and meat quality [23]. In our research, neither high-intensity (85–95 dB) nor low-intensity (65–75 dB) ventilation fan noise significantly improved the slaughter performance and meat quality of geese aged 21–70 days, even though the cortisol levels in the noise exposure group decreased. Of course, different research results have also been obtained in other animals. Research on pigs found that the concentration of α-linolenic acid (C18:3n-3) in loin and neck muscles decreased as the distance from a wind turbine increased [24]. Exposure to classical music (65–75 dB) over 42 days significantly lowered corticosterone levels and increased the a* value (measured at 24 h) in broiler breast muscle, while the L* and b* values (24 h) remained unaffected [19]. Therefore, the relationship between cortisol levels and meat quality may not be absolute.

### 4.3. Blood Stress Biomarkers and Antioxidant Indices

In general, stress activates the hypothalamic–pituitary–adrenal (HPA) axis, leading to the secretion of CORT from the adrenal gland [25] and resulting in growth suppression [26]. In the present study, geese exposed to prolonged noise exhibited significantly lower circulating ACTH and CORT concentrations than the controls, indicating that prolonged noise environments (65–75 dB and 85–95 dB) effectively reduced the physiological stress levels of geese, as they habituated to the long-term stable noise environment. One interpretation is that stable background noise functions as a form of environmental enrichment [27,28,29,30] and that a completely stimulus-deprived environment may not be optimal for rearing [31,32]. A moderate, steady acoustic “baseline” may also reduce responsiveness to minor, unpredictable disturbances (e.g., human movement or intermittent distant sounds). By dampening sensitivity to such perturbations, this acoustic context could lower hypothalamic–pituitary–adrenal (HPA) axis activation [33,34,35]. Furthermore, COR levels were significantly decreased in the low-noise group (65–75 dB) but numerically higher in the high-noise group (85–95 dB) compared with the control group. This differential response of COR to noise intensity suggests an intensity-dependent effect, which is generally consistent with previous findings in pullets [11].

T-AOC, GSH-Px, SOD, and CAT are all considered vital indicators of antioxidant status in the body; they can prevent oxidative stress by eliminating free radicals such as reactive oxygen species [36]. MDA has been recognized as a marker of oxidative stress and the final product of lipid peroxidation, which indirectly reflects the degree of cellular damage [37,38]. Oxidative stress can inhibit animal growth and lead to production loss. In the present study, no difference was observed in the plasma antioxidant capacity of geese with prolonged exposure to environmental noise. Hence, ventilation fan noise had no adverse effect on the antioxidant capacity and growth of geese.

### 4.4. Feeding Behavior

In the present study, we found that prolonged exposure to ventilation fan noise had no statistically significant effects on feeding behavior in geese aged 21–70 days. During the experiment, visual observation indicated that the geese in the noise treatment groups remained relatively stable, with no signs of panicked fleeing or aggressive pecking. In line with this observation, providing sound stimuli did not affect chick feeding behavior [39], a pattern consistent with prior work reporting limited or negligible effects of noise exposure on comparable outcomes in animals [40,41]. A plausible mechanism is habituation: when animals are repeatedly exposed to a stimulus without negative consequences, their behavioral and physiological responses may progressively attenuate [9,42,43]. In the present setting, ventilation fan noise constituted a continuous, stable, and predictable acoustic background; the flock may therefore have adapted early in the grow-out period, which could account for the absence of measurable disruptions in feeding behavior [44]. Chang et al. [11] also reported that long-term sound stimulation effectively mitigated the negative impact of transportation stress on pullets. Auditory stimulation through music can effectively mitigate aberrant behavior, alleviate anxiety, and enhance overall animal welfare [45,46].

## 5. Conclusions

Our results demonstrated that prolonged noise stimulation (65–75 dB and 85–95 dB) alleviated stress responses in commercial geese aged 21–70 days, without negatively affecting their growth performance, slaughter performance, meat quality, or feeding behavior. This suggests that geese can adapt to the rearing environment noise and provides a reference for developing stress-prevention feeding strategies in geese. Therefore, we recommend equipping large-scale goose houses with ventilation fans (noise range: 65–95 dB) during the summer.

## Figures and Tables

**Table 1:** Effects of ventilation fan noise level on the growth performance of geese from 21 to 70 days of age.

Variable ^1^	Treatment Group			SEM	*p*-Value
	Control Group	Low-Noise Group	High-Noise Group		
IBW (g/bird)	645.83	645.83	643.06	2.79	0.907
FBW (g/bird)	3239.44	3207.78	3377.50	49.40	0.349
ADG (g/bird/day)	52.93	52.41	56.18	1.12	0.350
ADFI (g/bird/day)	209.16	217.65	219.35	2.65	0.257
F/G (g/g)	3.97	4.18	3.91	0.07	0.267
TFI (g/)	61,031.67	63,056.67	63,331.67	709.82	0.374
Mortality (%)	2.78	5.56	2.78	1.68	0.761

^1^ Data are means of six replicates, with six geese per replicate. Abbreviations: IBW, initial body weight; FBW, final body weight; ADG, average daily gain; ADFI, average daily feed intake; F/G, feed/gain ratio; TFI, total feed intake.

**Table 2:** Effects of ventilation fan noise level on slaughter performance of geese at 70 days of age.

Variable ^1^	Treatment Group			SEM	*p*-Value
	Control Group	Low-Noise Group	High-Noise Group		
Dressing percentage (%)	87.22	87.16	88.37	0.26	0.088
Fully eviscerated yield (%)	73.48	72.77	73.91	0.30	0.320
Semi-eviscerated yield (%)	80.29	80.07	81.27	0.27	0.154
Breast muscle yield (%)	9.49	10.29	10.11	0.24	0.361
Leg muscle yield (%)	16.13	14.51	15.10	0.33	0.132
Skin-plus-subcutaneous-fat yield (%)	18.45	18.92	20.44	0.51	0.261
Abdominal fat yield (%)	2.03	2.24	2.57	0.21	0.188
Lean meat yield (%)	25.61	24.80	25.21	0.26	0.460

^1^ Data are means of six replicates, with one goose per replicate.

**Table 3:** Effects of ventilation fan noise level on meat quality of geese at 70 days of age.

Variable ^1^	Treatment Group			SEM	*p*-Value
	Control Group	Low-Noise Group	High-Noise Group		
Lightness (L*)	35.93	32.35	32.78	1.07	0.349
Redness (a*)	59.09	59.08	59.77	0.63	0.892
Yellowness (b*)	34.06	34.21	34.99	0.57	0.797
pH	5.79	5.73	5.78	0.03	0.695

^1^ Data are means of six replicates, with one goose per replicate.

**Table 4:** Effects of ventilation fan noise level on blood stress biomarkers of geese at 70 days of age.

Variable ^1^	Treatment Group			SEM	*p*-Value
	Control Group	Low-Noise Group	High-Noise Group		
ACTH (ng/L)	48.76 ^a^	36.45 ^b^	39.73 ^b^	1.65	0.001
CORT (ng/mL)	36.65 ^a^	25.8 ^c^	31.00 ^b^	1.31	<0.001
COR (ng/mL)	13.19 ^a^	9.69 ^b^	16.47 ^a^	0.90	0.002

^a, b, c^ In the same row, values with different lowercase letter superscripts are significantly different (*p* < 0.05), while those with common or no letter superscripts are not significantly different (*p* > 0.05). ^1^ Data are means of six replicates, with one goose per replicate. Abbreviations: ACTH, adrenocorticotropic hormone; CORT, corticosterone; COR, cortisol.

**Table 5:** Effects of ventilation fan noise level on blood antioxidant indices of geese at 70 days of age.

Variable ^1^	Treatment Group			SEM	*p*-Value
	Control Group	Low-Noise Group	High-Noise Group		
SOD (U/mL)	995.20	1101.52	1001.87	22.88	0.089
T-AOC (mmol/L)	0.71	0.76	0.74	0.01	0.349
CAT (U/mL)	0.17	0.19	0.22	0.01	0.188
GSH-PX (U/mL)	689.23	675.69	678.15	5.7	0.615
MDA (nmol/mL)	5.04	4.96	4.78	0.07	0.333

^1^ Data are means of six replicates, with one goose per replicate. Abbreviations: SOD, superoxide dismutase; T-AOC, total antioxidant capacity; CAT, catalase; GSH-PX, glutathione peroxidase; MDA, malondialdehyde.

**Table 6:** Effects of ventilation fan noise level on feeding behavior of geese from 21 to 70 days of age.

Variable ^1^	Treatment Group			SEM	*p*-Value
	Control Group	Low-Noise Group	High-Noise Group		
Feeding frequency (bouts/bird)	128.41	111.52	123.82	5.03	0.391
Feeding duration (s/bird)	642.23	557.58	619.09	25.17	0.391

^1^ Data are means of six replicates, with six geese per replicate. Feeding frequency, per-goose averages for feeding frequency during the daily observation period; feeding duration, per-goose averages for feeding duration during the daily observation period.

## Data Availability

All relevant data are within the manuscript.

## Abbreviations

The following abbreviations are used in this manuscript:

IBW	Initial body weight
FBW	Final body weight
ADFI	Average daily feed intake
ADG	Average daily gain
F/G	Feed/gain ratio
TFI	Total feed intake
ACTH	Adrenocorticotropic hormone
CORT	Corticosterone
COR	Cortisol
SOD	Superoxide dismutase
T-AOC	Total antioxidant capacity
CAT	Catalase
GSH-PX	Glutathione peroxidase
MDA	Malondialdehyde
